# Editorial: From genomics to antibiotic resistance in emerging pathogens

**DOI:** 10.3389/fmicb.2022.984301

**Published:** 2022-07-26

**Authors:** Ravi Kant, Annamari Heikinheimo, Tarja Sironen

**Affiliations:** ^1^Department of Virology, Faculty of Medicine, University of Helsinki, Helsinki, Finland; ^2^Department of Veterinary Biosciences, Faculty of Veterinary Medicine, University of Helsinki, Helsinki, Finland; ^3^Department of Food Hygiene and Environmental Health, Faculty of Veterinary Medicine, University of Helsinki, Helsinki, Finland; ^4^Unit of Microbiology, Finnish Food Authority, Seinäjoki, Finland

**Keywords:** antimicrobial resistance, emerging pathogens, genomics, emerging infectious disease, pathogens

Antimicrobial resistance (AMR) is one of the top 10 global public health threats facing the humanity. Main drivers of pathogenic microbes becoming resistant toward antimicrobials include misuse and overuse of antimicrobials resulting in the development of drug-resistant pathogens. Lack of inadequate infection prevention, clean water and sanitation further promotes the emergence and spread of AMR pathogens. In addition to constituting a health threat, AMR has economical and social dimensions. Overall, without urgent actions, AMR threatens us from achieving the United Nations Sustainable Development Goals.

In tackling AMR, new knowledge regarding identifying, characterizing, and tracking pathogens, their virulence genes, mobile genetic elements, and antimicrobial resistance genes is crucial. In addition to traditional molecular diagnostics and genotyping methods, next generation sequencing technologies have provided strong potential in deepening our understanding of the genomes, genomic epidemiology, and transmission routes of pathogenic microbes becoming resistant toward antimicrobials (Hendriksen et al., 2019). The use of new technologies in the research and routine public health practice such as the surveillance and control of pathogens is a rapidly expanding area. Special issues on specific topics are becoming a regular feature in scientific journals based on expertise of guest editors (Kant et al., 2020). In this special Issue our focus was on genomics of pathogens, especially the emerging and re-emerging pathogens and AMR associated with them. For our special issue, we received 17 manuscripts and, through rigorous review, selected 9 for publication (8 original articles and a review).

About half of the manuscript (4) in this special issue are focused on resistance associated with various *Escherichia coli* isolates. Kurittu et al. compared Extended Spectrum Betalactamase (ESBL)-producing *E. coli* isolates in Finland from different sources. The study described clinical isolates being genetically distinct from non-human sources and gave important information on global level of the spread of ESBL-producing *E. coli*. While, Liu et al. reported that the antibiotic resistance types of ARGs and suggested that *E. coli* is the primary antibiotic resistance reservoir of ARGs in CRC patients, providing valuable evidence for selecting appropriate antibiotics in the CRC treatment. Gonzalez et al. reported the resistance genes in 80 cefotaxime-resistant *E. coli* and 174 cefotaxime-resistant *K. pneumoniae* isolates from veal. They also followed-up the fecal carriage of ESBL-*K. pneumoniae* isolates from a subgroup of 9 animals and one animal carrying ESBL-*E. coli* to identify the clonal relatedness between the isolates. Leão et al. described the molecular epidemiology of the resistance genes and the blaCTX–M−65 genetic environment. Furthermore, they determined and described the genetic relatedness with other *E. coli* genomes for improved understandings into the public health impact of an ESBL producer seldom found in Europe.

In this special issue we also have manuscript focused on MRSA and ciprofloxacin resistant Salmonella. Sarkhoo et al. reports an upsurge in the predominance of the CC361-MRSA isolates with the dominance and transmission of a newly emerged ST672-MRSA [V/VT + fus] genotype in Kuwait hospitals. The CC361-MRSA isolates expressed resistance to different antibiotics including linezolid resistance reported for the first time in Kuwait. The discovery of the several virulence genes in these isolates and their isolation from different clinical samples signify their capacity to cause serious infections like other virulent MRSA lineages. While, Vázquez et al. characterizes ciprofloxacin resistant Salmonella Kentucky from Spanish hospitals and underlined the importance of continuous surveillance of the S. Kentucky ST198-CIPR clone.

Reslan et al. presented *Candida auris* isolates from different hospital units in Lebanon. This study disclosed the exclusivity of clade I lineage together with uniform resistance to fluconazole and amphotericin B in clinical *C. auris* isolates. Kong et al. investigated the genomic differences between a paired colistin-susceptible and -resistant *K. pneumoniae* isolates successively retrieved from a single patient, and confirmed the mechanism accountable for the emergence of high-level resistance to colistin during *in vivo* treatment and finally, McNeilly et al. highlights the antibacterial activities of NAg with its multi-targeting toxicity on *A. baumannii* and other bacteria. They also summarize the existing knowledge on the adaptive ability of *A. baumannii*, and other major bacterial species, to NAg and other silver agents. It is essential to recognize the applicability and long-term risks of the nanoparticle as a crucial alternative antimicrobial. The review also explains the emerging phenomenon of the metal-driven co-selection of antibiotic resistance to further stress the issue of overexposing bacteria to toxic heavy metals.

The articles in this special issue are focused on emerging and re-emerging pathogens and AMR associated with them. In our view, this is crucial for understanding AMR, and how this threat is currently developing. The findings in these articles could also contribute to the future development for diagnostics, therapies, and prevention tools to restrain and mitigate infectious disease threats and ensure sustainable, safe food and environment. We hope that these articles will help and stimulate readers working in the field of AMR.

## Author contributions

All authors contributed equally in writing this editorial. All authors contributed to the article and approved the submitted version.

## Conflict of interest

The authors declare that the research was conducted in the absence of any commercial or financial relationships that could be construed as a potential conflict of interest.

## Publisher's note

All claims expressed in this article are solely those of the authors and do not necessarily represent those of their affiliated organizations, or those of the publisher, the editors and the reviewers. Any product that may be evaluated in this article, or claim that may be made by its manufacturer, is not guaranteed or endorsed by the publisher.
